# Spatial Patterns of COVID-19 Vaccination Coverage by Social Vulnerability Index and Designated COVID-19 Vaccine Sites in Texas

**DOI:** 10.3390/vaccines10040574

**Published:** 2022-04-08

**Authors:** Dania Mofleh, Maha Almohamad, Ikponmwosa Osaghae, Sandra Bempah, Qianzi Zhang, Guillermo Tortolero, Ahmad Ebeidat, Ryan Ramphul, Shreela V. Sharma

**Affiliations:** 1 Department of Epidemiology, Human Genetics and Environmental Sciences, School of Public Health, University of Texas Health Science Center at Houston (UTHealth), Houston, TX 77030, USA; maha.almohamad@uth.tmc.edu (M.A.); ikponmwosa.osaghae@uth.tmc.edu (I.O.); guillermo.a.tortolero@uth.tmc.edu (G.T.); ryan.ramphul@uth.tmc.edu (R.R.); shreela.v.sharma@uth.tmc.edu (S.V.S.); 2 Geography Department, Kent State University, Kent, OH 44240, USA; sbempah@kent.edu; 3 Department of Management, Policy & Community Health, School of Public Health, University of Texas Health Science Center at Houston (UTHealth), Houston, TX 77030, USA; qianzi.zhang.1@uth.tmc.edu; 4 Department of Economics, Kellstadt Graduate School of Business, DePaul University, Chicago, IL 60604, USA; aebeidat@gmail.com

**Keywords:** COVID-19, vaccine, health inequities, GIS, vaccine coverage

## Abstract

Equitable access to the COVID-19 vaccine remains a public health priority. This study explores the association between ZIP Code–Tabulation Area level Social Vulnerability Indices (SVI) and COVID-19 vaccine coverage in Texas. A mixed-effects, multivariable, random-intercept negative binomial model was used to explore the association between ZIP Code–Tabulation Area level SVI and COVID-19 vaccination coverage stratified by the availability of a designated vaccine access site. Lower COVID-19 vaccine coverage was observed in ZIP codes with the highest overall SVIs (adjusted mean difference (aMD) = −13, 95% CI, −23.8 to −2.1, *p* < 0.01), socioeconomic characteristics theme (aMD = −16.6, 95% CI, −27.3 to −5.7, *p* = 0.01) and housing and transportation theme (aMD = −18.3, 95% CI, −29.6 to −7.1, *p* < 0.01) compared with the ZIP codes with the lowest SVI scores. The vaccine coverage was lower in ZIP Code–Tabulation Areas with higher median percentages of Hispanics (aMD = −3.3, 95% CI, −6.5 to −0.1, *p* = 0.04) and Blacks (aMD = −3.7, 95% CI, −6.4 to −1, *p* = 0.01). SVI negatively impacted COVID-19 vaccine coverage in Texas. Access to vaccine sites did not address disparities related to vaccine coverage among minority populations. These findings are relevant to guide the distribution of COVID-19 vaccines in regions with similar demographic and geospatial characteristics.

## 1. Introduction

Achieving an equitable approach to COVID-19 vaccine coverage remains a public health priority. In the United States, public health agencies prioritize the identification of high-risk communities to address inequalities in COVID-19 vaccine coverage and ensure equitable vaccine distribution [1]. This approach includes the evaluation of COVID-19 response according to race, ethnicity, geographic location, disability and other sociodemographic factors [1]. The Centers for Disease Control and Prevention (CDC) developed the Social Vulnerability Index (SVI) to help public health officials support the most vulnerable communities during a public health emergency [2]. Since the onset of the COVID-19 pandemic, the SVI has been used as a tool to monitor vaccine coverage equity and guide public health efforts to improve equitable COVID-19 vaccine coverage [3].

In efforts to address disparities among communities, the United States federal government collaborated with major pharmacies to increase accessibility to the COVID-19 vaccine [5,6]. The major driver of this effort was the notion that having access to a vaccine site could reduce the structural barriers related to transportation and health service location, increasing vaccine coverage [6,7,8]. The relationship between a vaccine access site and vaccine coverage has not been widely examined. This study aims to explore the association between the CDC SVI and COVID-19 vaccination coverage in locations with and without designated COVID-19 vaccine access sites, to investigate whether vaccines accessibility within a ZIP Code–Tabulation Area addresses inequities in COVID-19 vaccine coverage in the state of Texas as of 4 October 2021.

Vaccine coverage is defined as the percentage of eligible residents in a certain geographic area who received at least one dose of a COVID-19 vaccine approved by the Food and Drug Administration (FDA). Recent research showed COVID-19 vaccine coverage varied across communities by SVI theme [2,4]. An early study examining COVID-19 vaccine coverage from December 2020 to March 2021 showed counties with the highest overall social vulnerability index scores exhibited a 1.9% lower COVID-19 vaccine coverage rate compared with the least vulnerable counties [2]. A later study examining COVID-19 vaccine coverage from December 2020 to May 2021 found counties with the highest overall SVI scores exhibited 16.7% lower COVID-19 vaccine coverage compared with the least vulnerable counties in large metropolitan and non-metropolitan areas [4]. Findings from these earlier studies suggest disparities related to COVID-19 vaccine coverage increased over time and thus warrant further research [4].

## 2. Materials and Methods

We conducted an ecological study to assess the relationship between SVI and COVID-19 vaccination coverage in relation to designated COVID-19 vaccine access sites within each ZIP Code–Tabulation Area (ZCTA). We used publicly available datasets from the Texas Department of State Health Services [9], which provided the number of partially and fully vaccinated individuals in Texas by ZIP code and designated COVID-19 vaccine access site address locations. The physical addresses for designated COVID-19 vaccine sites included federal allocation centers, pharmacies, medical practices, community clinics, hospitals, vaccine hubs, local health departments, community vaccination centers and others. All data were aggregated at the ZCTA level using crosswalk files to standardize the units of analysis. For this paper, we refer to ZCTA as ZIP codes. The overall dataset contained 17,451,597 observations for individuals who had taken at least one dose of the COVID-19 vaccine, of which 109,198 (0.37% of the Texas population) had invalid or unknown ZIP code addresses and 258,725 (0.8%) were out-of-state. The analysis was limited to Texas boundaries with valid ZIP code addresses only. Shapefiles of Texas and ZIP code boundaries were obtained from the US Census TIGER/Line website.

### 2.1. Measures

#### 2.1.1. Dependent Variable

COVID-19 vaccine coverage is defined as the number of individuals who received either 1 or 2 doses of the Pfizer or Moderna vaccines or one dose of the Janssen vaccine, aggregated at the ZIP-code level. The primary outcome was operationalized as a percentage by calculating the number of COVID-19 vaccinated individuals within a ZIP code divided by the ZIP code population estimates for ages 10 years and older, as provided in the US Census 2019 American Community Survey 5-year estimate. Vaccination coverage was cumulatively calculated as of 4 October 2021.

#### 2.1.2. Independent Variables

CDC Social Vulnerability Index (SVI) for the State of Texas measures relative vulnerability within geographic areas [3]. The index ranks census tracts from 0 to 1 for all Texas census tracts based on 15 social factors categorized into one of four themes: socioeconomic characteristics; household composition and disability; minority status and language; and housing type and transportation [3]. A higher value for the SVI indicates a higher vulnerability. We divided each independent variable into quintiles, with quintile 1 (Q1) representing groups that are least vulnerable (lowest SVI quintile) and quintile 5 (Q5) representing groups that are most vulnerable (highest SVI quintile (Q5)).

Race/ethnicity population percentages were obtained from the US Census 2019 American Community Survey 5-year estimate by ZIP code and classified into non-Hispanic White, Hispanic or Latino, non-Hispanic Black or African American, non-Hispanic American Indian or Alaska Native, and non-Hispanic Asian American or Pacific Islander.

#### 2.1.3. Covariates

The following covariates obtained from the US Census 2019 American Community Survey 5-year estimate were assessed: gender percentage, Gini index coefficient (an index that measures income disparities), the median age of the population and the percentage of health workers. We used the Rural-Urban Commuting Area (RUCA) codes to identify rural and urban areas, obtained from the Department of Agriculture’s Economic Research Service (ERS) [10]. We used Texas RUCA codes by ZIP-code level and classified them into metropolitan areas (codes 1, 2 and 3), micropolitan areas (codes 4, 5 and 6), small towns (codes 7, 8 and 9) and rural areas (code 10). At the ZIP-code level, we stratified our analysis by vaccine–access site location (available vs. unavailable).

### 2.2. Statistical Analysis

The descriptive analysis included 1934 ZIP codes representing 99.7% of Texas ZIP codes. There were missing data not reported by the Texas Department of State Health Services for population density (*n* = 4), sex (*n* = 23), Gini index (*n* = 61), SVI (*n* = 3), percent of health-care providers (*n* = 1), ethnicity (*n* = 23), population median age (*n* = 14) and ZIP codes with less than five vaccine doses (*n* = 27). Available case analysis was used.

First, we used a sequential–sequential bivariate map to visualize the overall SVI against the vaccination coverage in Texas overlaid by designated vaccine–access site locations. We used a *t*-test or Mann–Whitney test to examine the differences between ZIP codes with unavailable designated COVID-19 vaccine access sites and ZIP codes with available designated COVID-19 vaccine sites.

Second, we used a mixed-effects, negative binomial regression model to examine our independent variables’ association with COVID-19 vaccination coverage. Using a crosswalk file, we aggregated the SVI to ZIP-code level and computed the weighted mean SVI using the overall population obtained from the US Census 2019 American Community Survey 5-year estimate. We rescaled the overall SVI and four social vulnerability themes by a multiple of 100. We dichotomized the 15 individual SVI components, gender percentage, and the race and ethnicity population percentages at the median, based on their distribution within ZIP codes. The likelihood-ratio test showed enough variability to favor a mixed-effects, negative binomial regression model over a negative binomial regression model. We conducted three separate mixed-negative binomial regression analyses with all three models including a random intercept for the county. Model 1 depicted overall ZIP codes, model 2 included ZIP codes without designated COVID-19 vaccine access sites, and model 3 included ZIP codes with a designated COVID-19 vaccine access sites. We estimated the adjusted mean difference for vaccination coverage between the most vulnerable quintile (Q5) and the least vulnerable quintile (Q1) for the overall SVI and four themes. Our final model adjusted for population density, the Gini index coefficient, percentage of health-care workers per ZIP code, population median age, and RUCA. The significance threshold was set at *p* < 0.05. We conducted all analyses using STATA 16.1 statistical software (StataCorp LLC, College Station, TX, USA) and ArcGIS Pro 2.8 (Environmental Systems Research Institute, Inc., Redlands, CA, USA).

## 3. Results

A total of 1934 ZIP codes were examined (99.7% of Texas ZIP codes). The results indicate that as of 4 October 2021, the average vaccination coverage in Texas ZIP codes was 67.9% (Table 1).

We found that ZIP codes with unavailable designated COVID-19 vaccine access sites exhibited statistically significantly lower average mean vaccine coverage (67.1% vs. 68.6%, *p* < 0.01), lower average population densities (60.5 residents/sq.-Mi vs. 701 residents/sq.-Mi; *p* < 0.01), lower average percentage of people living below the poverty line (14.8% vs. 16%, *p* < 0.01) and a lower percentage of the population who work in the health field (2.5% vs. 3.4%, *p* < 0.01) compared with ZIP codes with available designated COVID-19 vaccination access sites. Moreover, ZIP codes with unavailable designated COVID-19 vaccine access sites exhibited a higher percentage of rural ZIP codes (20.1% vs. 4.0%, *p* < 0.01), higher percentage of small-town ZIP codes (14.3% vs. 9.7%, *p* < 0.01), higher average percentage of people aged 65 years and older (18.3% vs. 14%, *p* > 0.01) and higher average percentage of individuals with a disability (16.3% vs. 13.1%, *p* < 0.01) compared with ZIP codes with available designated COVID-19 vaccine access sites (Table 1).

**Figure 1:**
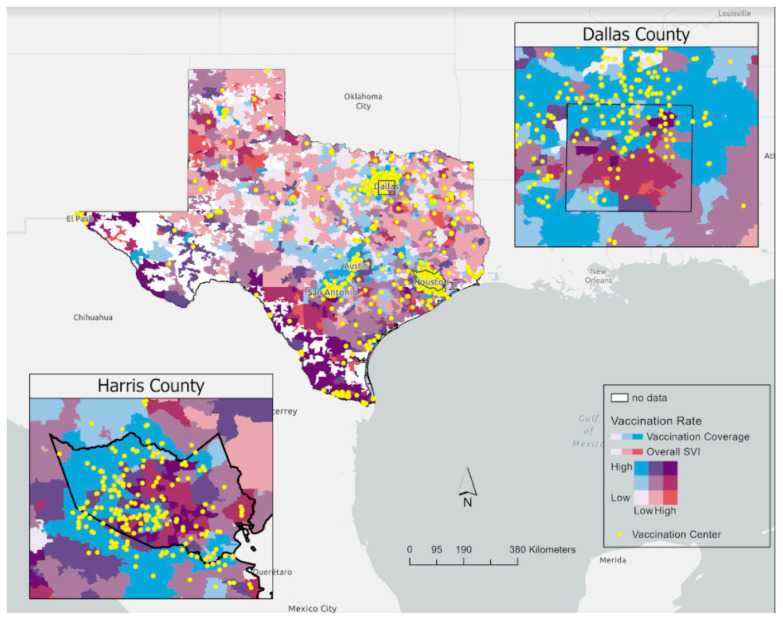
A sequential–sequential bivariate map to visualize the overall SVI against the vaccination coverage in Texas overlaid by vaccination center location. The color of the ZIP codes indicates the relationship between vaccination coverage and overall SVI ranking, where the red color indicates ZIP codes with higher scores for overall SVI and low vaccine-uptake rates. In contrast, the blue color represents ZIP codes with high vaccine coverage and low overall SVI. The yellow dots represent the vaccination centers.

Figure 1 displays a sequential–sequential bivariate map to visualize the overall SVI by the vaccination coverage rate per 100 population in Texas. Figure 1 also presents a small-scale bivariate map for Harris and Dallas Counties with the surrounding areas, implying that ZIP codes with overall high SVI scores have low-to-moderate vaccine coverage. A higher rate of vaccination coverage and higher number of vaccination centers are mostly populated in highly dense cities like Dallas, Houston, Austin, San Antonio, and El Paso. In contrast, less densely populated areas across Texas had fewer vaccination centers, lower vaccination coverage rates and mostly moderate overall SVI scores.

Results of adjusted mixed-negative binomial regression analyses (Table 2) among the overall population (Model 1) showed mean vaccine coverage was significantly higher in the ZIP codes with the lowest SVI scores (least vulnerable) (72%) compared with ZIP codes with the highest overall SVI score (59%) (adjusted mean difference (aMD) = −14.8, 95% CI, −26.2 to −3.5, *p* = 0.02). Vaccine coverage was significantly lower in ZIP codes with the highest SVI scores (most vulnerable) for the socioeconomic characteristics theme (aMD = −16.6, 95% CI, −27.3 to −5.7, *p* = 0.01) and for the housing and transportation theme (aMD = −18.3, 95% CI, −29.6 to −7.1, *p* < 0.01) compared with ZIP codes with the lowest SVI scores (least vulnerable) (Table 2).

In our stratified analysis, ZIP codes without a designated COVID-19 vaccine access site (Model 2) showed that the socioeconomic characteristics theme, household and disability theme, and the racial/ethnic minority status and language theme were negatively associated with vaccine coverage. A higher mean vaccine coverage was shown among ZIP codes with the lowest SVI scores for the socioeconomic characteristics theme (aMD = −27.3, 95% CI, −49.8 to −4.9, *p* = 0.03), the household and disability theme, (aMD = −41.8, 95% CI, −72.8 to −10.8, *p* = 0.01) and the racial/ethnic minority status and language theme (aMD = −22.3, 95% CI, −43.8 to −0.9, *p* = 0.04) compared with ZIP codes with the highest SVI scores for each theme, respectively.

Among ZIP codes with available designated COVID-19 vaccine access sites (model 3), the negative association between the socioeconomic characteristics theme and vaccine coverage persisted (aMD = −15.7, 95% CI, −27.1 to −4.2, *p* = 0.01). However, there was no significant association between the household and disability theme and vaccine coverage. Furthermore, we found that ZIP codes with the lowest SVI scores exhibited significantly higher vaccine coverage compared with ZIP codes with the highest SVI scores with regard to overall SVI (aMD = −20.5, 95% CI, −32 to −9, *p* < 0.01), the socioeconomic theme (aMD = −15.7, 95% CI, −27.1 to −4.2, *p* < 0.01) and the housing and transportation theme (aMD = −17.7, 95% CI = −30.4 to −5, *p* = 0.01).

Results from Table 3 show the mean vaccine coverage was significantly higher in ZIP codes with poverty levels at or below the median (63.5%) compared with ZIP codes above the median (59.4%) (aMD = −4.1, 95% CI, −7.1 to −1.1, *p* = 0.01). These differences persisted in areas without a designated COVID-19 vaccine access site (aMD = −6.5, 95% CI, −11.7 to −1.2, *p* = 0.02) and in ZIP codes with an available designated vaccine site (aMD = −3.8, 95% CI= −7.2 to −0.4, *p* = 0.03).

We also found differences in vaccine coverage related to race and ethnicity. Vaccine coverage was significantly higher in ZIP codes with below the median percentages of Hispanic or Latino populations (67%) compared with ZIP codes with above the median percentages of Hispanic or Latino populations (63.7%) (aMD = −3.3, 95% CI, −6.5 to −0.1, *p* = 0.04) and ZIP codes with a percentage of Black or African American populations below the median exhibited statistically higher vaccine coverage (63.1%) compared with ZIP codes with above the median percentages of Black or African American populations (59.3%) (aMD = −3.7, 95% CI, −6.4 to −1, *p* = 0.01). The adjusted mean difference in vaccine coverage among the Black or African American population was higher in ZIP codes without a designated COVID-19 vaccine access site (aMD = −9.3, 95% CI, −14.3 to −4.3, *p* < 0.01) and lower in ZIP codes with designated vaccine sites (aMD = −3.5, 5% CI, −6.4 to −0.6, *p* = 0.02). The American Indian or Alaska Native population displayed similar trends for vaccine coverage across ZIP codes without designated vaccine sites (aMD = −6.5, 95% CI, −11.4 to −1.6, *p* = 0.01) and ZIP codes with designated vaccine sites (aMD = −5.6, 95% CI, −7.7 to −2.3, *p* < 0.01). Vaccine coverage was also significantly higher in ZIP codes with below the median percentages of Asian American or Pacific Islander populations (63%) compared with ZIP codes with above the median percentages of Asian American or Pacific Islander populations (60.3%) (aMD = −2.7, 95% CI, −5.4 to 0, *p* = 0.05). The difference in vaccine coverage among Asian Americans or Pacific Islanders persisted in ZIP codes without a designated vaccine access site (aMD = −5.2, 95% CI, −10.2 to −0.2, *p* = 0.04); however, the difference was not significant in ZIP codes with a designated vaccine site.

## 4. Discussion

Since the rollout of the first COVID-19 vaccine in late 2020, the focus has been on ensuring equitable vaccine coverage and guaranteeing the most vulnerable populations and regions are not overlooked [2]. Texas is the second largest state in the US [11] and ranks 6th as the most diverse state in terms of minority populations, [12] comprised of about 40% Hispanic and 12% Black non-Hispanic ethnicities [13]. Over 30% of Texas residents live below 200% of the federal poverty level [14], thus presenting an opportunity to examine vaccine coverage in a highly diverse region ecologically.

Our results were consistent with the previous literature reporting lower vaccine coverage among adults living in poverty and having less education [4]. Studies evaluating COVID-19 vaccine hesitancy prior to the COVID-19 vaccine rollout suggested that socioeconomically vulnerable individuals reported a lower likelihood of receiving the COVID-19 vaccine [15,16]. Along with previous findings, our study highlights the need to identify barriers to equitable vaccine coverage among socioeconomically vulnerable populations [17]. The lower vaccination coverage in the ZIP codes with the highest scores for the housing and transportation theme raises concerns about persistent structural barriers such as transportation cost [18]. Moreover, the ZIP codes with designated COVID-19 vaccine access sites are more prevalent in metropolitan areas, suggesting the need to further investigate vaccine-site access points, such as the association with their distance from public transportation and highly vulnerable populations [19].

In ZIP codes without a designated COVID-19 vaccine access site, the household and disability theme was associated with substantial vaccine coverage disparities between the least and most vulnerable ZIP codes. Observed disparities could be explained by the higher mean percentage of the population aged 65 years and older living in areas without a designated vaccine access site, i.e., those most likely to face challenges related to COVID-19 vaccine access [20]. Findings from this study were consistent with previous studies reporting lower COVID-19 vaccine coverage in areas with a higher percentage of elderly adults with social vulnerabilities [21,22], which raises a concern about vaccine center accessibility among the elderly population, and addressing such structural barriers should be of utmost priority. Adopting innovative approaches to ensure equitable vaccine access, such as the in-house vaccination program implemented in Texas in mid-March 2021, could close existing coverage gaps [23]. In addition, the provision of transportation arrangements to vaccination appointments could increase vaccine uptake in ZIP codes with low coverage [18].

We also observed differences in vaccine coverage related to the racial/ethnic minority status and language theme in ZIP codes without a designated COVID-19 vaccine access site. The results were not surprising since previously published reports from Texas observed fewer vaccine access sites within minority neighborhoods [24]. Differences in vaccine coverage among the Black population were consistent across ZIP codes with and without a designated COVID-19 vaccine access site. In contrast, differences in COVID-19 vaccine coverage in the Hispanic or Latino population were observed in ZIP codes with a designated COVID-19 vaccine access site. Our study agrees with a previously published study and suggests that the availability of vaccine access sites to the minority population may not be adequate to address vaccine inequities. Findings are indicative of underlying barriers [8] which could be explained by technological barriers [25] as well as challenges related to language and communication barriers [26]. These results imply the need for outreach efforts in highly dense regions of minority populations.

Our study is relevant to current efforts to improve COVID-19 vaccine coverage in the state of Texas, across the US and other global regions with similar demographics and geospatial characteristics to our study. As such, this investigation can be used to guide interventions to increase vaccine coverage rates in the most vulnerable regions. However, our study had some limitations. We included persons aged 10–12 years in our calculation of vaccination rates due to data availability. At the time of this analysis, COVID-19 vaccines were only approved under emergency-use authorization by the FDA and CDC for ages 12 years and older [27]. As such, findings from this study may underestimate the true effect estimates. Our study is subject to residual confounding due to data availability limitations.

Additionally, missing address-level data for records could have contributed to information bias and affected the measure-of-association estimates. This study is prone to misclassification bias due to changes in COVID-19 vaccine supply throughout the study period, creating a COVID-19 vaccination desert in areas within ZIP codes with a designated vaccine access site. Finally, this ecological study may be subject to ecological fallacy as results may not reflect individual-level experience.

## 5. Conclusions

SVI negatively impacted COVID-19 vaccine coverage in Texas. Our findings suggest that the availability of designated COVID-19 vaccine access sites within a ZIP code may be necessary but insufficient to address vaccine coverage inequities associated with socioeconomic, housing and transportation, and racial/ethnic vulnerabilities. Further attention should be given to minority groups to address existing structural accessibility to the COVID-19 vaccine through policy and community interventions.

## Figures and Tables

**Table 1:** Characteristics of ZIP codes included in the study by vaccine access site availability.

	Overall (*n* = 1934)		Vaccine Access Site Unavailable (*n* = 976)		Vaccine Access Site Available (*n* = 958)		*p*-Value
	Mean	SD	Mean	SD	Mean	SD	
**COVID-19 Coverage Rate (per 100 people)**	67.9	53.9	67.1	68.7	68.6	32.9	0.00
**Geographic Characteristic** ^b^							
Population Density (residents/sq.-mi)	378.6	799.1	60.5	271.5	701	1002.6	0.00
**Sex Characteristics** ^b^							
Women, %	49.7	7.4	49.2	9.6	50.1	4.1	0.00
Men, %	50.3	7.4	50.8	9.6	49.9	4.1	0.00
**Socioeconomic Characteristics** ^b^							
Percentage Living Below Poverty Line ^a^	15.4	8.3	14.8	7.8	16.0	8.7	0.01
Percentage Unemployment Rate ^a^	5.5	2.9	5.4	3.2	5.6	2.5	0.01
Percentage Per Capita Income ^a^	28,509.2	11,149.4	26,755.3	7415.0	30,288.7	13,728.5	0.00
Percentage With No High School Diploma ^a^	17.9	10.0	18.0	9.1	17.8	10.9	0.07
Gini Index for Income Disparities	4.2	0.8	4.0	0.9	4.4	0.5	0.00
**Household Composition and Disability Characteristics** ^b^							
Percentage of People Aged 65 and Older ^a^	16.2	6.1	18.3	6.3	14.0	5.2	0.00
Percentage of People Aged 17 and Younger ^a^	23.8	5.1	23.2	4.9	24.5	5.2	0.00
Percentage With a Disability ^a^	14.7	5.0	16.3	4.9	13.1	4.6	0.00
Percentage of Single-parent Households ^a^	8.5	4.1	7.5	4.0	9.5	4.0	0.00
**Racial and Ethnic Characteristics** ^b^							
Percentage White	55.1	28.0	63.9	27.9	46.4	25.3	0.00
Percentage Minority (all persons except non-Hispanic whites) ^a^	44.8	24.9	37.5	22.8	52.1	24.7	0.00
Percentage Hispanic, %	32.2	26.8	27.3	27.1	37.1	25.5	0.00
Percentage Black, %	8.5	12.9	6.1	12.5	10.9	12.8	0.00
Percentage American Indian or Alaska Native	0.3	0.9	0.4	1.2	0.3	0.4	0.00
Percentage Asian American or Pacific Islander	2.2	4.6	0.8	2.5	3.6	5.7	0.00
Percentage of People Who Speak English “Less than Well” ^a^	5.5	6.2	4.4	5.5	6.7	6.8	0.00
**Housing Type and Transportation Characteristics** ^b^							
Percentage of Housing in Structures With 10 or More Units ^a^	7.6	13.5	2.6	7.6	12.7	16.1	0.00
Percentage of Mobile Homes ^a^	10.5	29.8	15.4	39.5	5.5	39.4	0.00
Percentage of Occupied Housing Units With More People Than Rooms ^a^	4.3	3.1	3.9	2.9	4.7	3.3	0.00
Percentage of Households With No Vehicle Available ^a^	5.0	4.0	4.2	3.3	5.7	4.4	0.00
Percentage of People in Institutionalized Group Quarters ^a^	3.4	7.9	3.8	9.1	3.0	6.5	0.00
**Occupation**							
Percentage of Population With Health Occupation	2.9	3.4	2.5	4.2	3.4	2.4	0.00
	**N**	%	**N**	%	**N**	%	
**Rural-Urban Commuting Area, %**							
Metropolitan Area	1193	61.7	463	47.4	730	76.4	0.00
Micropolitan Area	263	13.6	177	18.1	86	9	
Small Town	233	12.1	140	14.3	93	9.7	
Rural Area	243	12.6	196	20.1	47	4.9	

Abbreviation, SD = standard deviation; N = number; % = percentage. ^a^ Components of the Social Vulnerability Index themes. ^b^ Missing data for the population density (*n* = 4), sex (*n* = 23), Gini Index (*n* = 61), Social Vulnerability Index themes (*n* = 3), ethnicity for White, Hispanic, Black, American Indian or Alaska Native and Asian American or Pacific Islander (*n* = 23), percentage of population in health-care professions (*n* = 1), and Rural-Urban Commuting Area (*n* = 2).

**Table 2:** Association differences in COVID-19 vaccine coverage between the least (Q1) and most (Q5) vulnerable ZIP codes stratified by the availability of a designated COVID-19 vaccine access site ^a^.

	(Model 1) Overall ZIP Codes				(Model 2) Vaccine Access Site Unavailable				(Model 3) Vaccine Access Site Available			
Variable	Adjusted Mean	Adjusted Mean Differences	95% CI		Adjusted Mean	Adjusted Mean Differences	95% CI		Adjusted Mean	Adjusted Mean Differences	95% CI	
Overall SVI												
Q1 (least vulnerable)	72				67				79.3			
Q5 (Most vulnerable)	59	−13	−23.8	−2.1	62.8	−4.3	−26.5	17.9	58.8	−20.5	−32	−9
Socioeconomic Theme												
Q1 (least vulnerable)	73.5				79.7				76.2			
Q5 (most vulnerable)	56.9	−16.6	−27.4	−5.7	52.4	−27.3	−49.8	−4.9	60.5	−15.7	−27.1	−4.2
Household and Disability Characteristics Theme												
Q1 (least vulnerable)	71.8				96.7				69.7			
Q5 (most vulnerable)	64.8	−7	−18.2	4.1	54.9	−41.8	−72.8	−10.8	70.3	0.6	−10.7	11.9
Racial/Ethnic Minority Status and Language Theme												
Q1 (least vulnerable)	63.4				68.7				68.3			
Q5 (most vulnerable)	53.7	−9.7	−20.5	1	46.4	−22.3	−43.8	−0.9	59.7	8.7	−19.8	2.5
Housing and Transportation Theme												
Q1 (least vulnerable)	74.3				65.8				77.4			
Q5 (most vulnerable)	56	−18.3	−29.6	−7.1	59.9	−6	−26.5	14.5	59.7	−17.7	−30.4	−5

Abbreviations: Q1, quintile 1; Q5, quintile 5; 95% CI, 95% confidence interval. ^a^ Results from a mixed, multivariable, random intercept negative binomial model adjusted for population density, the Gini index coefficient, percentage of health-care workers per ZIP code, population median age, and Rural-Urban Commuting Area. Vaccination coverage is defined as the number of individuals who received either 1 or 2 doses of the Pfizer or Moderna vaccines or 1 dose of the Jansen vaccine.

**Table 3:** The differences in COVID-19 vaccine coverage between most vulnerable (above median) and least vulnerable (below median) ZIP codes by designated COVID-19 vaccine access site availability ^a^.

	(Model 1) Overall ZIP Codes				(Model 2) Vaccine Access Site Unavailable				(Model 3) Vaccine Access Site Available			
Variable	Adjusted Mean Vaccine Coverage	Adjusted Mean Differences	95% CI		Adjusted Mean Vaccine Coverage	Adjusted Mean Differences	95% CI		Adjusted Mean Vaccine Coverage	Adjusted Mean Differences	95% CI	
**Gender Characteristics**												
Percentage Women (median at 50.4%)												
Below Median	62.2				61.6				65.0			
Above Median	60.8	−1.5	−3.9	0.9	58.8	−2.8	−7.7	2.2	63.3	−1.7	−4.3	0.9
Percentage Men (median at 49.6)												
Below Median	60.6				58.9				63.1			
Above Median	62.5	1.9	−0.5	4.3	61.5	2.7	−2.3	7.6	65.3	2.2	−0.4	4.9
**Socioeconomic Characteristics**												
Percentage Living Below Poverty Line, (median at 13.8%) ^b^												
Below Median	63.5				63.5				66.0			
Above Median	59.4	−4.1	−7.1	−1.1	57.1	−6.5	−11.7	−1.2	62.3	−3.8	−7.2	−0.4
Unemployment Rate, % (median at 5.1%) ^b^												
Below Median	64.3				63.2				65.6			
Above Median	58.8	−5.5	−8.4	−2.7	57.4	−5.8	−11.0	−0.6	62.6	−3.0	−6.1	0.1
Per Capita Income, (median at 26,437.6 USD) ^b^												
Below Median	62.6				61.9				65.0			
Above Median	61.1	−1.5	−4.2	1.2	58.7	−3.2	−8.4	2.1	63.9	−1.2	−4.2	1.8
Percentage with No High School Diploma, (median at 15.8%) ^b^												
Below Median	61.5				61.1				64.79			
Above Median	61.5	0.0	−3.1	3.1	59.6	−1.5	−6.8	3.8	63.50	−1.3	−4.8	2.3
**Household Composition and Disability Characteristics**												
Percentage of People Aged 65 and Older, (median at 15.7%) ^b^												
Below Median	62.8				66.1				64.7			
Above Median	61.4	−1.5	−4.9	2.0	57.9	−8.2	−14.6	−1.8	64.4	−0.3	−4.1	3.5
Percentage of People Aged 17 and Younger, (median at 24.1%) ^b^												
Below Median	58.7				60.2				60.2			
Above Median	64.9	6.2	3.1	0.2	60.2	0.0	−5.9	5.9	67.6	7.4	4.1	10.7
Percentage with a Disability, (median at 14.5%) ^b^												
Below Median	63.3				65.1				64.9			
Above Median	60.3	−3.0	−6.6	0.6	57.8	−7.3	−13.5	−1.2	63.3	−1.6	−5.7	2.5
Percentage of Single-parent Households, (median at 7.7%) ^b^												
Below Median	60.6				60.9				63.3			
Above Median	62.2	1.6	−1.6	4.7	59.6	−1.3	−7.3	4.7	64.4	1.1	−2.3	4.4
**Racial/Ethnic Minority Status and Language**												
Non-Hispanic White, (median at 59.3%)												
Below Median	62.7				58.2				65.4			
Above Median	61.7	−1.0	−4.6	2.7	62.4	4.2	−3.0	11.3	66.2	0.9	−2.9	4.6
Percentage Minority (all people except non-Hispanic whites), (median at 39.1%)												
Below Median	61.4				61.2				66.4			
Above Median	63.3	1.9	−1.7	5.5	60.1	−1.1	−7.8	5.6	65.4	−0.9	−4.7	2.8
Hispanic or Latino, (median at 23.7%)												
Below Median	63.9				61.1				67.0			
Above Median	59.5	−2.5	5.6	0.6	59.8	−1.2	−7.2	4.7	63.7	−3.3	−6.5	−0.1
Non-Hispanic Black or African American, (median at 3.6%)												
Below Median	63.1				63.5				66.0			
Above Median	59.3	−3.7	−6.4	−1.0	54.1	−9.3	−14.3	−4.3	62.5	−3.5	−6.4	−0.6
Non-Hispanic American Indian or Alaska Native, (median at 0.1%)												
Below Median	63.5				62.0				67.2			
Above Median	58.6	−4.9	−7.2	−2.6	55.9	−6.1	−10.9	−1.4	61.6	−5.6	−8.2	−2.9
Non-Hispanic Asian American or Pacific Islander, (median at 0.4%)												
Below Median	63.0				61.5				64.8			
Above Median	60.3	−2.7	−5.4	0.0	56.3	−5.2	−10.2	−0.2	63.9	−0.9	−3.8	2.1
Percentage of People Who Speak English “Less than Well”, (median at 3.2%)												
Below Median	61.7				61.3				64.8			
Above Median	61.5	−0.2	−2.8	2.5	59.3	2.0	−7.0	3.1	65.0	0.2	−2.6	2.9
**Housing Type and Transportation Characteristics**												
Percentage of Housing in Structures With 10 or More Units, (median at 1.8%) ^b^												
Below Median	61.3				60.8				60.7			
Above Median	64.8	3.5	0.3	6.6	59.6	−1.1	−6.2	3.9	67.8	7.1	3.8	10.4
Percentage of Mobile Homes, (median at 14.7%) ^b^												
Below Median	63.3				60.1				66.5			
Above Median	60.2	−3.1	−6.1	−0.2	60.2	0.2	−4.5	4.9	60.3	−6.3	−9.3	−3.2
Percentage of Occupied Housing Units With More People Than Rooms, (median at 3.6%) ^b^												
Below Median	62.4				61.8				64.7			
Above Median	60.6	−1.7	−4.6	1.1	58.9	−2.8	−8.2	2.6	63.8	−0.9	−4.0	2.1
Percentage of Households With No Vehicle Available, (median at 4%) ^b^												
Below Median	62.0				59.8				65.9			
Above Median	61.4	−0.7	−3.3	1.9	61.2	1.4	−3.6	6.4	63.3	−2.7	−5.6	0.3
Percentage of People in Institutionalized Group Quarters, (median at 0.8%) ^b^												
Below Median	62.9				60.9				66.7			
Above Median	60.2	−2.7	−5.0	−0.4	59.5	−1.4	−5.8	2.9	61.8	−4.9	−7.4	−2.4

^a^ Results from a mixed, multivariable, random-intercept negative binomial model adjusted for population density, Gini index coefficient, and percent of health-care workers per ZIP code. ^b^ Components of the Social Vulnerability Index themes. Vaccination coverage is defined as the number of individuals who received either 1 or 2 doses of the Pfizer or Moderna vaccines or 1 dose of the Jansen vaccine.

## Data Availability

Publicly available datasets were analyzed in this study. This data can be found at https://www.dshs.texas.gov/coronavirus/additionaldata/ (accessed on 10 October 2021).
